# Editorial: Digital mental health: Interventions and assessment

**DOI:** 10.3389/fpsyg.2022.1014527

**Published:** 2022-11-24

**Authors:** Cristina Costescu, Ioana R. Podina, Alexandra Voinescu

**Affiliations:** ^1^Department of Special Education, Babeş-Bolyai University, Cluj-Napoca, Romania; ^2^Laboratory of Cognitive Clinical Sciences, University of Bucharest, Bucharest, Romania; ^3^Department of Applied Psychology and Psychotherapy, University of Bucharest, Bucharest, Romania; ^4^Department of Psychology, University of Bath, Bath, United Kingdom

**Keywords:** digital mental health, digital interventions, digital assessment, well-being, psychological distress

Robotic, web-based, virtual reality (VR), or mobile-based digital mental health interventions (DMHI) offer tremendous potential to promote mental health and well-being across a range of age groups (Neguţ et al., 2016; Aboujaoude et al., 2020; Voinescu et al., 2021). More than 70% of young people use digital devices, but their mental health problems remain under-diagnosed and under-treated (UNICEF, 2018; World Health Organization, 2020). Therefore, DMHIs could provide excellent opportunities to expand access to diagnostic and intervention services, as well as to improve empowerment, participation, help-seeking, and essential resources to address the stigma associated with mental health (Kaushik et al., 2016; Freeman et al., 2022). In addition, interventions designed to enhance psychological well-being may lessen the negative consequences of the COVID-19 epidemic (De Kock et al., 2022). Several investigations during the COVID-19 epidemic in the community showed a strong negative correlation between psychological well-being and anxiety, despair, and distress (see meta-analysis Salari et al., 2020). DMHIs may improve accessibility, can bridge social gaps, and allow users to log in anonymously whenever they want (Sorkin et al., 2021).

Because DMHIs are believed to be an effective tool to address underutilization of professional mental health services (Garrido et al., 2019), several studies assessed their effectiveness. Overall, these studies supported the effectiveness of DMHIs in reducing anxiety and depression symptoms and showed that digital mental health programs can be as effective as in-person programs and sometimes more effective for adults (Cuijpers et al., 2010; Prescott et al., 2022) and children (Pǎsǎrelu et al., 2017). Several reviews of technology applications for mental health found positive outcomes and important benefits (Firth et al., 2017; Garrido et al., 2019). However, more research is required for vulnerable populations such as older adults with mental health problems, as they are at high risk of being excluded from DMHI due to a number of factors such as lack of knowledge and support in using digital tools (Seifert et al., 2019). Another problem with using DMHIs among the general population is that the dropout rates are very high (Torous et al., 2018).

It is important to take into account some of the drawbacks of DMHI, such as how well these interventions might work for severe mental health conditions (such as psychosis, bipolar disorder, or personality disorders) (Bucci et al., 2018). Potential validity issues and level of accessibility are also major challenges. As in other research domains with a focus on human machine interfaces such as designing computer interfaces for older adults or people with special needs, the need to use adaptable and usable interfaces is highly recognized (Fisk et al., 2004; Morgan et al., 2017). The main drawback of using new technologies is that they may be too difficult to use, that could lead to a decrease in user engagement. This is due to the new types of technologies used to deliver the interventions and the rapid technological improvements (Borghouts et al., 2020). Other drawbacks and potential barriers to the use of DMHIs include data privacy, data storage, and user confidentiality (Lustgarten et al., 2020).

In this issue, the studies address some of the limitations discussed above and aims to suggestr solutions and future directions.

## The session wants and need outcome measure: The development of a brief outcome measure for single-sessions of web-based support

The paper developed by de Ossorno Garcia et al. described the planning, development, and usability evaluation of the Brief Outcome Measure for Single-Sessions of Web-Based Support, an online web-based mental health platform for children and young people (Kooth). A key implication of the study conducted by de Ossorno Garcia et al. concerns the importance of engaging various categories of end users (e.g., experts, practitioners and students that access the service) during the developmental stage of the SWAN-ON. This approach highlighted the importance of applying well-known co-design approaches currently used in the development of e-health interventions (Thabrew et al., 2018) to the developmental stage of an instrument that measures the effects of digital interventions such as the SWAN-ON.

## Investigating the persuasive effects of testimonials on the acceptance of digital stress management trainings among university students and underlying mechanisms: A randomized controlled trial

In this experimental study, Apolinário-Hagen et al. investigated the effect of various features of narrative information in supplemented testimonials on the acceptance of digital mental health services (digi-MHSs) among students. Overall, results showed that adding testimonials to written information was associated with higher intentions to use and more positive attitudes toward digi-MHSs for stress prevention. No significant effects of testimonials were identified for the attitudes toward online interventions for stress coping or therapy. A significant mediation effect of source credibility on attitudes revealed that student testimonials were more credible than those by experts, but no significant mediation effect was observed for intentions to use.

## A randomized controlled trial of clinician-guided internet-based cognitive behavioral therapy for depressed patients in singapore

The study conducted by Lu et al. investigated if a clinician-guided internet-based Cognitive Behavioral Therapy (iCBT) programme is effective in improving symptoms of depression, anxiety, psychological distress and functional impairment among outpatients with depression in a psychiatric hospital in Singapore. The study consisted of two arms: the iCBT and a waiting list control group. The iCBT intervention used a blended approach with three face-to-face sessions and six online sessions. Results showed positive and significant effects in improving depressive symptoms. This study was among the first studies conducted in Singapore and included other samples than the Western culture. Its significant results support the effectiveness of using an iCBT intervention in Asian culture.

## Case report: Feasibility of a novel virtual reality-based intervention for patients with schizophrenia

Vass et al. described a case report study with a focus on a novel immersive VR-based intervention designed to reduce symptoms associated with schizophrenia. A 50-year-old person with a diagnosis of schizophrenia took part in the study. The 9-week VR-based intervention (1 h/session/week) took place in an individual setting and was led by a licensed clinical psychologist under the supervision of an experienced psychotherapist. Several outcomes were measured before, after, and at follow-up: mood and emotions, pragmatic language skills and quality-of-life and theory of mind-related outcomes. Authors reported improvements at post-test and follow-up on theory of mind and communicative-pragmatic skills.

## Developing a brief tele-psychotherapy model for COVID-19 patients, and their family-members

Biagianti et al. focused on the development of a brief tele-psychotherapy model for COVID-19 patients and their family members. The intervention was based on a literature review on the topic of psychotherapeutic interventions for COVID-19-related symptoms. The program consisted of eight individual weekly remote sessions (50 min each) that will be delivered using secure video conference software. The paper is part of a larger project with the aim to evaluate acceptability, feasibility, efficacy, and effectiveness of the proposed program. In the context of social and participatory restrictions and with the aim of increasing accessibility to psychological interventions for people who live in geographical zones without an easy access to these services, remote tele-psychotherapy would be of great impact.

## Innovating technology-enhanced interventions for youth suicide: Insights for measuring implementation outcomes

In a review, Szlyk et al. analyzed 12 randomized clinical trials (RCTs), that assess the efficacy and effectiveness of technology-enhanced suicide interventions. Their aim was to explain how technology-enhanced interventions for youth suicide can be categorized using behavioral intervention technologies and how implementation outcomes can be measured in future effectiveness trials. The results showed increased heterogeneity due to the technology provider and support, and its content. The results also suggested that outcomes defined in the continuum of behavioral interventional technology can raise considerations for how outcomes could be integrated in more implementation focused studies.

## Access to nature *via* virtual reality: A mini-review

The paper published by Li et al. described the results of a mini-review that included 15 studies, to answer questions concerning the benefits of using virtual nature and if VR exposure in VR produces similar effects with traditional media for nature scenes. Their results showed that the benefits from virtual nature are generally consistent with those from exposure to real nature, meaning that current VR app can facilitate a restorative natural environment with positive psychological outcomes.

## Keeping connected with school: Implementing telepresence robots to improve the well-being of adolescent cancer patients

The work developed by Powell et al. aimed to improve well-being of adolescent cancer patients by using telepresence robots. They interviewed 47 participants with various vulnerable participants such as: adolescent with cancer, healthcare professionals, schoolteachers, and parents of adolescents with cancer. The aim of the first study was to investigate the benefits, acceptability, barriers, and enablers of utilizing robots in schools for adolescents with cancer. In the second phase of their study, they interviewed the participants about their experience in using a robot to enable adolescents to attend school remotely. In conclusion, the social and academic connections facilitated by the robot improved the mood of the adolescents and reduced their feelings of stress and loneliness.

## Can we boost treatment adherence to an online transdiagnostic intervention by adding self-enhancement strategies? Results from a randomized controlled non-inferiority trial

Isbăşoiu et al. focused on comparing two intervention strategies for decreasing symptoms of anxiety and depression and increasing the self-parameters. They compared the UP intervention for transdiagnostic treatment of anxiety and affective disorders that was developed by Barlow et al. (2011) and contain 9 modules web-based with a Self-enhanced 9UP version with enhancement strategies that address self-concepts. All primary outcomes that target anxiety and depression symptoms and secondary outcomes that address the self-concept were administrated online at pre-treatment, post-treatment, and 6 months follow-up. 284 participants with at least one clinical disorder were randomly allocated to one of the two conditions. Overall, both groups produced significant increases in both primary and secondary outcomes in post-test and at 6-months follow-up, however, there were no significant differences between the two groups neither in primary and secondary outcomes nor in terms of treatment adherence.

## Research on the method of depression detection by single-channel electroencephalography sensor

Lei et al. developed a model to detect depressive mood based on the attention and mediation signals produced by a single channel EEG headset. They hypothesized that when the participants have depression an M-shaped pattern appears in their attention and/or mediation signals. They recruited 158 senior high school students, for their first study and respectively 73 and 69 participants aged 18–60 for the second and third studies. They compared the results gained through EEG headset with other psychological tests that measured depressive symptoms, such as PHQ9, Hamilton Depression Rating Scale, and House-Tree-Person drawing test. Results showed that the consistency rate of the two methods was 61.4% in the first study, 63.38% in the second study, and 91.3% in the third study.

## Making a virtue out of necessity: COVID-19 as a catalyst for applying internet-based psychological interventions for informal caregivers

A compelling opinion piece was also included in the current special issue. According to Simonella et al. the COVID-19 pandemic was a glass half empty, but also a glass half full situation. The negative consequences were frequently acknowledged, but the unforeseen positive effects were less discussed. One such significant advantage was a decrease in resistance to using digital technologies. The authors argue that the world must use this opportunity right away to create and extensively disseminate internet-based treatments to those in need. Their opinion piece exemplifies the benefits of internet-based interventions for informal caregivers, family members, neighbors, friends, or other non-kin and who provide unpaid assistance to someone who is disabled, frail, or ill.

## Remote vs. in-person delivery of LearningRx one-on-one cognitive training during the COVID-19 pandemic: A noninferiority study

One other study that capitalized on the remote online delivery of interventions during the COVID-19 pandemic was conducted by Lawson Moore et al.'s study. Three hundred eighty-one children and adults received cognitivetraining (ThinkRx) from 18 cognitive training facilities. One group received the cognitive training face-to-face, whereas another received it *via* Zoom teleconferencing. In terms of total IQ score, processing speed, fluid reasoning, long-term memory, and visual processing, remote delivery was comparable to the in-person delivery. The authors concluded that delivering cognitive training remotely may represent a good substitute for in-person instruction.

## Efficacy of an ACT and compassion-based eHealth program for self-management of chronic pain (iACTwithPain): Study protocol for a randomized controlled trial

In a study by Carvalho et al., Acceptance and Commitment Therapy (ACT) was designed to be part of an e-Health program for the self-management of chronic pain. Given that ACT is frequently used to treat trauma and recover from mental health problems, testing its effectiveness for chronic pain is represents a novel attempt, particularly in a digital setting. The efficacy of the study will be assessed at baseline, post-intervention, and 3- and 6-month follow-up with treatment as usual, ACT-only intervention, and an iACTwithPain + self-compassion as arms of the trial. Outcomes such as the impact of pain, subjective discomfort, and quality of life are still being tested in the trial.

## A self-applied multi-component psychological online intervention based on UX, for the prevention of complicated grief disorder in the mexican population during the COVID-19 outbreak: Protocol of a randomized clinical trial

The current issue also includes protocol research by Dominguez-Rodriguez et al.. Through a multifaceted online intervention that combines components of ACT, CBT, Mindfulness, and Positive Psychology, the study protocol focused on investigating complicated grief. The intervention was ompared with a a waitlist control group using an RCT design with a-priori power size computations, pre-post efficacy assessment accompanied by a 3- and 6-month follow-up. As before, the article is a worthwhile read for those interested both in the design as well as the components of the proposed intervention.

## Engagement in digital mental health interventions: Can monetary incentives help?

Because of the high dropout rates, engagement with DMHIs is a serious concern. Few studies focused on engagement factors that can boost involvement with DMHIs, such as financial incentives, according to Boucher et al.. The authors questioned whether monetary incentives (MI) are a workable tactic for increasing user engagement with DMHIs. Therefore, the article begun with a review of the literature and then presented a pilot research where they assessed the effects of different degrees of MI on user engagement. In short, results suggested that monetary incentives were effective “only on technological engagement” when interventions are spread out over multiple sessions. There was no one-size-fits-all-method to increase involvement since “more money does not necessarily imply more benefit” and “there is large variability in how people respond to MI.”

## Screening social anxiety in adolescents through the eyes of their careers

Additionally, psychometric research written by Garcia-Lopez et al. was included in the present special issue. The parent version of “The Social Phobia and Anxiety Inventory, Brief form” (SPAIB), was administered online, was assessed for its psychometric qualities. The sample consisted of 179 parents and legal guardians of children. Results showed good factor structure, internal consistency, and construct validity. As a result, parents now have a valid screening tool for their children's social anxiety that remains underdiagnosed. Nonetheless, future studies should test SPAIB's psychometric properties on other samples.

## Remote assessment of depression using digital biomarkers from cognitive tasks

The authors of this paper used three standard cognitive tasks (D2 Test of Attention, Delayed Matching to Sample Task, and Spatial Working Memory Task) where people with depression are known to perform differently than healthy people. The performance of the users on these tasks was used to predict depression scores as measured with Patient Health Questionnaire (PHQ-9). These tests were made available online in a series of two trials. Results supported the use of the model with all three tests compared to using them individually.

## The development of explicit and implicit game-based digital behavioral markers for the assessment of social anxiety

The current special issue also contained a study on behavioral biomarkers, that targeted social anxiety. Dechant et al. argued that one extract of digital behavioral markers from game-based behavior and use them to assess social anxiety. Digital distance from other game characters was one such marker that mimics the real-life behavior of a socially anxious person. The study's findings suggested that social anxiety's hallmark behaviors can be seen in the world of video games. Higher levels of social anxiety led to altered movement patterns toward non-playing characters (NPCs). There were several other interesting results highlighted in the paper and including cueing how game-based digital behavioral markers can be used in the future for the assessment and screening of social anxiety.

## The therapeutic goals set by university students in an anonymous web-based therapy and support setting

Another interesting study using descriptive student data from the Kooth Student web-based therapy platform is that of Hanley et al.. The goal of the study was to extract the characteristics common to students using web-based therapy services, the therapeutic goals students typically set for themselves, and how well they are progressing toward those goals. The study analyzed anonymous data from 211 students who reported their goals on the platform. The most common goals expressed by students focused on getting additional support in exploring emotions. Some other findings indicate that women are more likely to achieve their goals, while students regardless of gender are more likely to seek help than self-help. The article also offers some insights into the barriers to collecting meaningful outcome data from anonymous services.

## Conclusion and discussions

The research from this special issue addressed most of the issues concerning effectiveness of the DMHI. First, it's important to consider user-related factors including demographics (age, sex, education, and socioeconomic status), diagnosis and severity of symptoms, attitudes toward the use of digital technologies, personality features, and the availability of treatment. Secondly, the level of assistance the users require and its adaptability.

Thirdly, the technological characteristics of the devices and platforms used (i.e., the costs of the device, the necessary resources to make it functional, and technical issues/problems the user may encounter) and privacy and data collection are also important assets.

Although there are thousands of studies that supported the effectiveness of DMHI, this special issue showed that research may focus more on examining the attitudes of different users toward technology and digital content. In addition, the practice of co-designing technology-mediated interventions, where the users collaborate with the developers, has emerged as a current need in several studies. Another concept wort exploring in other studies concerns the importance of cultural differences and their effects on the effectiveness of DMHI. We could also compare the effectiveness of different types of technologies ns, such as computer-generated scenarios vs. 360° videos, different designs of content interactivity, and VR vs. 2D content. Particularly given challenges posed by COVID, research adapted rapidly and addressed important and challenging issues concerning well-being and offered potentially effective solutions such as remote interventions delivered with DMHI.

In the current special issue, most studies used various DMHI platforms ranging from traditional technical devices, web-based content, smartphones, to digital tablets. However, there were also studies that used immersive solutions such as VR or telepresence, robots and wearable devices. VR-based solutions that use machine learning and algorithms and wearable technologies, are notable prospects that could be included in future studies. Although many of the studies in this special issue analyzed the effects of DMHI on mood disorders, distress, or psychological well-being, two studies described the use of DMHI for schizophrenia and suicidal ideation (Szlyk et al.; Vass et al.).

We also mention the importance of involving multiple stakeholders in the therapeutic process (e.g., teachers or parents), and the adaptability of the program to suit the needs of the participants as important issues. One study attempted to address one of the main problems in this area, treatment adherence, using self-enhancement methods (Isbăşoiu et al.). Previous studies by Zarski et al. (2018) suggested that self-regulatory effort was a predictor of treatment adherence, so the use of self-enhancement techniques could be a valid way to increase treatment adherence. However, no significant results were found. Another study (Boucher et al.) examined whether financial incentives could increase user engagement. Although it discussed several studies, it concluded that monetary incentives can only be effective in increasing engagement in the use of the technology, but not necessary in the delivery of the treatment, that usually lasts several sessions. Taken together, results suggest that treatment adherence and user engagement are key issues that need further study.

While two of the studies in this special issue focused on the use of DMHI for assessment of cognitive processes; the majority were intervention oriented. However, more research is needed to determine the effectiveness and precision of digital evaluation tools especially delivered *via* telepresence (e.g., remote and automatized neuropsychological assessment). The next generation of evaluation methods for emotional symptoms mayd be developed using new technologies such as VR, social robots, or wearable devices. One advantage of using immersive solutions would be the ability to quantify physiological symptoms more accurately and objectively in real time. For psychological interventions, all of the research in this special issue included intervention strategies from different therapeutic paradigms.

Overall, current issue offers unique insights into the use of DMH assessments and interventions. Although several studies support the efficacy of DMHIs in reducing symptoms associated with psychological disorders, there are several important issues to consider when developing interventions that aim to reduce psychological distress. Co-designing digital interventions with end users, increasing user motivation and engagement, and considering the cost and ease of use of the technology used, are only some of the factors that should be pondered in. Future research could examine treatment adherence and accessibility of DMHIs for across a wide range of population ranging from people with less prevalent mental disorders (e.g., personality disorders, psychosis) and people from disadvantaged backgrounds and other cultures than Western culture. In addition, future studies could evaluate the impact of different technological tools and tailor them to the specific population they are most appropriate for.

## Author contributions

CC, IP, and AV wrote and revised the manuscript. All authors contributed to the article and approved the submitted version.

## Conflict of interest

The authors declare that the research was conducted in the absence of any commercial or financial relationships that could be construed as a potential conflict of interest.

## Publisher's note

All claims expressed in this article are solely those of the authors and do not necessarily represent those of their affiliated organizations, or those of the publisher, the editors and the reviewers. Any product that may be evaluated in this article, or claim that may be made by its manufacturer, is not guaranteed or endorsed by the publisher.

## References

[B1] Aboujaoude E. Gega L. Parish M. B. Hilty D. M. (2020). Editorial: digital interventions in mental health: current status and future directions. Front. Psychiatry 11, 111. 10.3389/fpsyt.2020.00111 32174858 PMC7056878

[B2] Barlow D. H. Ellard K. K. Fairholme C. P. Farchione T. J. Boisseau C. L. Allen L. B.. (2011). The Unified Protocol for Transdiagnostic Treatment Of Emotional Disorders: Client Workbook. Oxford: Oxford UniversityPress.

[B3] Borghouts J. Eikey E. Mark G. De Leon C. Scheller S. Schneider M.. (2020). Barriers and facilitators to user engagement with digital mental health interventions: a systematic review. J. Med. Internet Res. 23, e24387. 10.2196/preprints.24387 33759801 PMC8074985

[B4] Bucci S. Lewis S. Ainsworth J. Haddock G. Machin M. Berry K.. (2018). Digital interventions in severe mental health problems: lessons from the Actissist development and trial. World Psychiatry 17, 230. 10.1002/wps.20535 29856569 PMC5980566

[B5] Cuijpers P. Donker T. van Straten A. Li J. Andersson G. (2010). Is guided self-help as effective as face-to-face psychotherapy for depression and anxiety disorders? A systematic review and meta-analysis of comparative outcome studies. Psychological medicine 40, 1943–1957. 10.1017/S0033291710000772 20406528

[B6] De Kock J. H. Latham H. A. Cowden R. G. (2022). The mental health of healthcare workers during the COVID-19 pandemic: a narrative review. Curr. Opin. Psychiatry 35, 311–316. 10.1097/YCO.0000000000000805 35855506

[B7] Firth J. Torous J. Nicholas J. Carney R. Pratap A. Rosenbaum S.. (2017). The efficacy of smartphone-based mental health interventions for depressive symptoms: a meta-analysis of randomized controlled trials. World Psychiatry 16, 287–298. 10.1002/wps.20472 28941113 PMC5608852

[B8] Fisk D. Charness N. Czaja S. J. Rogers W. A. Sharit J. (2004). Designing for Older Adults. Boca Raton, FL: CRC Press.

[B9] Freeman D. Lambe S. Kabir T. Petit A. Rosebrock L. Yu L.. (2022). Automated virtual reality (VR) therapy (gameChange) treating agoraphobic avoidance and distress in patients with psychosis: a multicentre, parallel group, single-blind, randomised controlled trial in England with mediation and moderation analyses. Lancet Psychiatry 9, 375–388. 10.1016/S2215-0366(22)00060-8 35395204 PMC9010306

[B10] Garrido S. Millington C. Cheers D. Boydell K. Schubert E. Meade T.. (2019). What works and what doesn't work? A systematic review of digital mental health interventions for depression and anxiety in young people. Front. Psychiatry 10, 759. 10.3389/fpsyt.2019.00759 31798468 PMC6865844

[B11] Kaushik A. Kostaki E. Kyriakopoulos M. (2016). The stigma of mental illness in children and adolescents: a systematic review. Psychiatry Res. 243, 469–494. 10.1016/j.psychres.2016.04.042 27517643

[B12] Lustgarten S. D. Garrison Y. L. Sinnard M. T. Flynn A. W. (2020). Digital privacy in mental healthcare: current issues and recommendations for technology use. Curr. Opin. Psychol. 36, 25–31. 10.1016/j.copsyc.2020.03.012 32361651 PMC7195295

[B13] Morgan P. L. Voinescu A. Williams C. Caleb-Solly P. Alford C. Shergold I.. (2017). “An emerging framework to inform effective design of human-machine interfaces for older adults using connected autonomous vehicles,” in International Conference on Applied Human Factors and Ergonomics (Cham: Springer), 325–334.

[B14] Neguţ A. Matu S. A. Sava F. A. David D. (2016). Virtual reality measures in neuropsychological assessment: a meta-analytic review. Clin. Neuropsychol. 30, 165–184. 10.1080/13854046.2016.1144793 26923937

[B15] Pǎsǎrelu C. R. Andersson G. Bergman Nordgren L. Dobrean A. (2017). Internet-delivered transdiagnostic and tailored cognitive behavioral therapy for anxiety and depression: a systematic review and meta-analysis of randomized controlled trials. Cogn. Behav. Ther. 46, 1–28. 10.1080/16506073.2016.1231219 27712544

[B16] Prescott M. R. Sagui-Henson S. J. Chamberlain C. E. Castro Sweet C. Altman M. (2022). Real world effectiveness of digital mental health services during the COVID-19 pandemic. PLoS ONE 17, e0272162. 10.1371/journal.pone.0272162 35980879 PMC9387818

[B17] Salari N. Hosseinian-Far A. Jalali R. Vaisi-Raygani A. Rasoulpoor S. Mohammadi M.. (2020). Prevalence of stress, anxiety, depression among the general population during the COVID-19 pandemic: a systematic review and meta-analysis. Glob. Health 16, 1–11. 10.1186/s12992-020-00589-w 32631403 PMC7338126

[B18] Seifert A. Reinwand D. A. Schlomann A. (2019). Designing and using digital mental health interventions for older adults: being aware of digital inequality. Front. Psychiatry 10, 568. 10.3389/fpsyt.2019.00568 31447716 PMC6696744

[B19] Sorkin D. H. Janio E. A. Eikey E. V. Schneider M. Davis K. Schueller S. M.. (2021). Rise in use of digital mental health tools and technologies in the United States during the COVID-19 pandemic: survey study. J. Med. Internet Res. 23, e26994. 10.2196/26994 33822737 PMC8054774

[B20] Thabrew H. Fleming T. Hetrick S. Merry S. (2018). Co-design of eHealth interventions with children and young people. Front. Psychiatry 9, 481. 10.3389/fpsyt.2018.00481 30405450 PMC6200840

[B21] Torous J. Nicholas J. Larsen M. E. Firth J. Christensen H. (2018). Clinical review of user engagement with mental health smartphone apps: evidence, theory and improvements. Evid. Based Ment. Health 21, 116–119. 10.1136/eb-2018-102891 29871870 PMC10270395

[B22] UNICEF. (2018). Operational Guidelines on Community Based Mental Health and Psychosocial Support in Humanitarian Settings: Three-Tiered Support for Children and Families. New York, NY: UNICEF.

[B23] Voinescu A. Petrini K. Stanton Fraser D. Lazarovicz R. A. Papavă I. Fodor L. A.. (2021). The effectiveness of a virtual reality attention task to predict depression and anxiety in comparison with current clinical measures. Virtual Reality 1–22. 10.1007/s10055-021-00520-7

[B24] World Health Organization. (2020). Mental Health and Psychosocial Considerations During the COVID-19 Outbreak. World Health Organization.

[B25] Zarski A. Berking M. Reis D. Lehr D. Buntrock C. Schwarzer R.. (2018). Turning good intentions into actions by using the health action process approach to predict adherence to internet-based depression prevention: secondary analysis of a randomized controlled trial. J. Med. Internet Res. 20, e9. 10.2196/jmir.8814 29326097 PMC5785685

